# Identification and Validation of Novel Long Non-coding RNA Biomarkers for Early Diagnosis of Oral Squamous Cell Carcinoma

**DOI:** 10.3389/fbioe.2020.00256

**Published:** 2020-04-15

**Authors:** Yue Li, Xiaofang Cao, Hao Li

**Affiliations:** ^1^Department of Orthodontics, The Second Affiliated Hospital of Harbin Medical University, Harbin, China; ^2^Department of Endodontics, The Second Affiliated Hospital of Endodontics, Harbin, China; ^3^Department of Stomatology, The Fourth Affiliated Hospital of Heilongjiang University of Traditional Chinese Medicine, Harbin, China

**Keywords:** biomarker, diagnosis, long non-coding RNAs, oral squamous cell carcinoma, epigenetic

## Abstract

Long non-coding RNAs (lncRNAs) are recently emerging as a novel promising biomarker for cancer diagnosis and prognosis. Despite these previous investigations, the expression pattern and diagnostic role of lncRNAs in oral squamous cell carcinoma (OSCC) remain unclear. In this study, we identified six novel lncRNA biomarkers (*LINC01697*, *LINC02487*, *LOC105376575*, *AC005083.1*, *SLC8A1-AS1*, and *U62317.1*) from a list of 29 differentially expressed lncRNAs using the least absolute shrinkage and selection operator (LASSO) method in the discovery dataset of 167 OSCC samples and 45 normal oral tissues. Using the logistic regression method, we constructed a six lncRNAs-based diagnostic risk model (6lncRNAScore) which was able to differentiate between OSCC samples and control samples with high performance with AUC of 0.995 and high diagnostic specificity of 88.9% and sensitivity of 98.2% in the discovery dataset. The diagnostic performance of the 6lncRNAScore was further validated in another two independent OSCC dataset with AUC of 0.968 and 1.0. Functional enrichment analysis for lncRNA biomarkers-related mRNAs suggested that lncRNAs biomarkers tended to be involved in the lipid metabolic process. Together, our study highlighted the importance of lncRNAs in OSCC and demonstrated the utility of lncRNA expression as a promising biomarker for early diagnosis of OSCC.

## Introduction

Oral cavity and pharynx cancer are one of ten leading cancer types with approximately 53,000 estimated new cases and 10,860 estimated deaths in the United States according to cancer statistics, 2019 (Siegel et al., 2019). Oral squamous cell carcinoma (OSCC) is the most common oral cavity and pharynx cancer accounting for more than 90% of oral cancers (Tandon et al., 2017). Despite the improvement in therapeutic approaches, surgery followed by postoperative radiation or chemoradiation is still standard treatment. The prognosis of OSCC patients remains unfavorable with 5-year survival rates of 40–50% in large part due to diagnosis in advanced stages. Therefore, identifying biomarkers for early diagnosis is a crucial way to improve the survival rate and quality of life of OSCC patients.

Recent advances in high-throughput sequencing theologies have found that the approximately 2% human genome encodes only ∼20,000 protein-coding genes and the vast majority of the human genome were actively transcribed as non-coding RNAs (ncRNAs) (Wright and Bruford, 2011). Long nc RNAs (lncRNAs) are a newly discovered class of ncRNAs ranging in length from 200 nt to ∼100 kilobases (kb) (Ulitsky and Bartel, 2013). Functional studies in cell lines and animal models have demonstrated that lncRNAs have emerged as key genomic regulators in a wide variety of biological pathways including differentiation and development (Rinn and Chang, 2012; Batista and Chang, 2013). A large number of dysregulated lncRNAs have been discovered in multiple tumor types compared with the corresponding normal tissues, which demonstrated the important roles of lncRNAs in cancer development, progression, and treatment (Zhang et al., 2013; Li et al., 2017). Increasing evidence have suggested that lncRNAs may be promising biomarkers in cancer diagnosis and prognosis compared with protein-coding genes because lncRNAs are expressed in a more highly cell type-, tissue-, and disease type-specific manner than protein-coding genes and their expression may be a better indicator of the tumor status (Hauptman and Glavac, 2013; Zhou et al., 2015, 2017; Salviano-Silva et al., 2018; Bao et al., 2019).

In this study, to evaluate the potential of lncRNAs biomarkers in the diagnosis of OSCC, we compared differential expression profiles of lncRNAs between OSCC samples and controls in a larger OSCC dataset. Then we used multiple statistical methods to identify novel lncRNA biomarkers and developed an lncRNA-based diagnostic prediction model, which was validated in several independent OSCC datasets.

## Materials and Methods

### OSCC Datasets

We retrospectively collected a total of 262 samples from three publicly available datasets from the Gene Expression Omnibus (GEO) database,^1^ including 167 OSCC samples and 45 normal oral tissues from GSE30784 dataset^2^ (Chen et al., 2008), 26 OSCC samples and 12 control samples from GSE9844 dataset^3^ (Ye et al., 2008), and six OSCC samples and six adjacent non-involved oral tissue from GSE74530 dataset^4^ (Oghumu et al., 2016). The largest sample dataset GSE30784 was used as a discovery dataset and the other two datasets were used as independent testing datasets.

### Acquisition and Analysis of lncRNA Expression Profiles

The raw microarray data files (.CEL files) of three OSCC sample datasets on Affymetrix Human Genome U133 Plus 2.0 (Affymetrix HG-U133 Plus 2.0) were downloaded directly from the GEO database and were processed and normalized using robust multichip average method by R “affy” package. LncRNA expression data of all samples in three datasets were retrieved by repurposing the probes based on the NetAffx annotation files of the probe sets and the annotation files of RefSeq and GENCODE according to previous studies (Zhou et al., 2018, 2019). Finally, expression data of 2466 lncRNAs of three OSCC datasets were obtained for further analysis.

Differential expression analysis of lncRNAs between OSCC samples and control samples was performed using the R package “limma” (version 3.42.0), those lncRNAs with | log2(fold change)| > 1 and false discovery rate (FDR) adjusted *p*-value < 0.05 was considered as differentially expressed lncRNAs.

### Construction of lncRNA-Based Diagnostic Risk Model for Early Detection of OSCC

The least absolute shrinkage and selection operator (LASSO) method was used to select the most useful predictive features from the list of differentially expressed lncRNAs as diagnostic lncRNA biomarkers in the discovery dataset. Then all diagnostic lncRNA biomarkers were fitted a logistic regression model as the covariates, and an lncRNA-based diagnostic risk model was constructed by using the sum of expression value of lncRNA biomarkers weighted by the unbiased coefficients estimates from the logistic regression model (Hao et al., 2017) as follows:

$$\mathrm{Diagnostic}\,\mathrm{risk}\,\mathrm{score}=\frac{e^{w^{T}*x+b}}{1+e^{w^{T}*x+b}}$$

Where *x* is expression levels of each lncRNA biomarker, *w* is the coefficients estimates from the logistic regression model, and *b* is 18.35146902. The risk score range from 0 to 1, and >0.5 was used as the cutoff for the diagnosis of OSCC.

The diagnostic performance of the lncRNA-based diagnostic risk model was evaluated by a receiver operating characteristic (ROC) curve and the area under the ROC (AUC).

### Functional Enrichment Analysis

Functional enrichment analysis of GO and Kyoto encyclopedia of genes and genomes (KEGG) pathway was performed to identify significantly enriched GO terms and KEGG pathways of mRNAs correlated with diagnostic lncRNA biomarkers using the R package “clusterprofiler” (Yu et al., 2012).

## Results

### Analysis of Altered lncRNA Expression Pattern in OSCC

**FIGURE 1:**
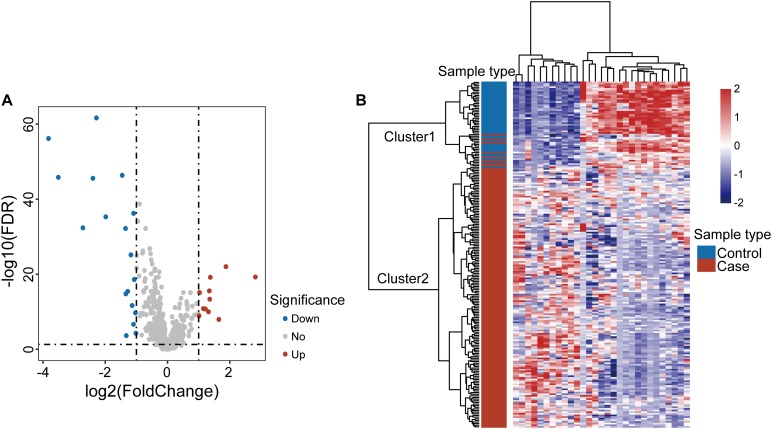
Analysis of differentially expressed genes in oral squamous cell carcinoma. **(A)** Volcano plot displaying differential expressed lncRNAs between OSCC samples and control samples. **(B)** Unsupervised clustering of all samples based on the expression pattern of 29 differentially expressed lncRNAs in the discovery dataset.

To explore the altered lncRNA expression pattern in OSCC, we compared lncRNA expression profiles between 167 OSCC samples and 45 control samples in the discovery dataset and identified 29 differentially expressed lncRNAs [| log2(fold change)| > 1 and FDR-adjusted *p*-value < 0.05] (Supplementary Table S1). Of them, 18 lncRNAs are downregulated and 11 lncRNAs are upregulated in OSCC compared with control samples (Figure 1A). Results of unsupervised hierarchical clustering analysis showed that all samples in the discovery dataset can could be grouped into two clusters based on the expression pattern of these 29 differentially expressed lncRNAs. 159 of 167 OSCC samples and one control were classified into Cluster 2, and 44 of 45 control samples and eight OSCC samples were classified into Cluster 1. Statistical analysis indicated a significant association between clusters and disease status (*p* < 0.001, chi-squared test) (Figure 1B).

### Development and Validation of the lncRNA-Based Diagnostic Risk Model for Early Detection of OSCC in the Discovery Dataset

**FIGURE 2:**
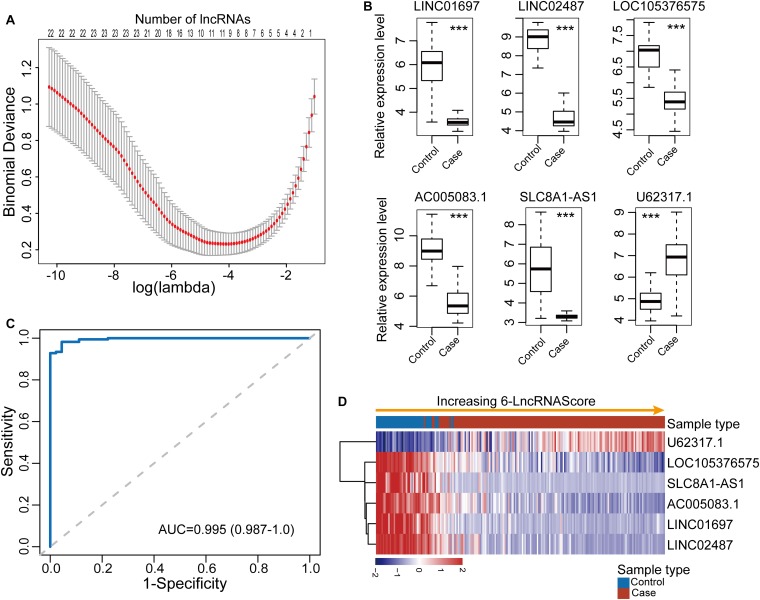
Development and performance evaluation of the lncRNA-based diagnostic risk model in the discovery dataset. **(A)** Feature selection for lncRNA biomarkers using the least absolute shrinkage and selection operator (LASSO) method. **(B)** Expression patterns of six lncRNA biomarkers between OSCC samples and control samples. **(C)** Receiver operating characteristic (ROC) curves for 6lncRNAScore. **(D)** Expression heatmap of six lncRNA biomarkers of samples with increasing 6lncRNAScore. ***p < 0.001.

To identify diagnostic lncRNA biomarkers, 29 differentially expressed lncRNAs were analyzed using the LASSO with 10-fold cross-validation and the turning parameter λ of 0.055. As shown in Figure 2A, we obtained six lncRNAs (*LINC01697*, *LINC02487*, *LOC105376575*, *AC005083.1*, *SLC8A1-AS1*, and *U62317.1*) from the list of differentially expressed lncRNAs as optimal diagnostic biomarkers considering a balance between classification accuracy and the number of lncRNAs (Table 1). Of six lncRNA biomarkers, five lncRNAs biomarkers (*LINC01697*, *LINC02487*, *LOC105376575*, *AC005083.1*, and *SLC8A1-AS1*) seem to be tumor suppressors and downregulated in OSCC, while lncRNA U62317.1 tended to be an oncogene and up-regulated in OSCC (Figure 2B). Using a logistic regression method, a six-lncRNAs-based diagnostic risk model was generated (named 6lncRNAScore). When the 6lncRNAScore was applied to samples of the discovery dataset, the 6lncRNAScore correctly classified 164 of 167 OSCC samples and 40 of 45 control samples, achieving an AUC of 0.995 with the sensitivity of 98.2% and the specificity of 88.9% (Figures 2C,D).

**TABLE 1:** Detailed information of six diagnostic lncRNA biomarkers identified in the discovery dataset.

Ensemble id	Gene name	Genomic location	Coefficient^a^	FDR *p*-value^b^
ENSG00000232079	LINC01697	Chr 21: 28,048,404-28,137,611 (+)	−0.0953	<0.001
ENSG00000203688	LINC02487	Chr 6: 167,679,626-167,696,290 (−)	−0.4317	<0.001
105376575	LOC105376575	NC_000011.10 (17332042.17349973)	−1.8052	<0.001
ENSG00000233834	AC005083.1	Chr 7: 20,217,577-20,221,700 (+)	−0.8215	<0.001
ENSG00000227028	SLC8A1-AS1	Chr 2: 39,786,453-40,255,209 (+)	−0.4150	<0.001
ENSG00000272666	U62317.1	Chr 22: 50,542,305-50,542,906 (−)	0.8571	<0.001

*^*a*^Derived from the logistic regression model. ^*b*^Derived from differential expression analysis.*

### Independent Validation of the 6lncRNAScore in Two Independent OSCC Datasets

**FIGURE 3:**
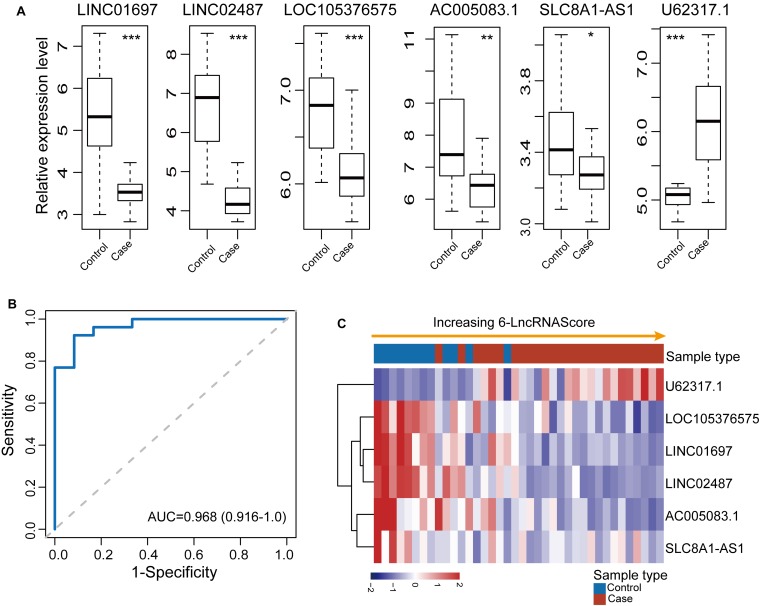
Independent validation of the 6lncRNAScore in 38 samples of GSE9844. **(A)** Expression patterns of six lncRNA biomarkers between OSCC samples and control samples. **(B)** Receiver operating characteristic (ROC) curves for 6lncRNAScore. **(C)** Expression heatmap of six lncRNA biomarkers of samples with increasing 6lncRNAScore. *p < 0.05, **p < 0.01, ***p < 0.001.

To confirm the robustness of the 6lncRNAScore for early diagnosis, the 6lncRNAScore was tested in an additional independent OSCC dataset (GSE9844) of 38 samples. We first investigated the expression patterns of six lncRNA biomarkers between OSCC samples and control samples in the GSE9844 dataset. As shown in Figure 3A, five lncRNA biomarkers (*LINC01697*, *LINC02487*, *LOC105376575*, *AC005083.1*, and *SLC8A1-AS1*) were significantly downregulated and one lncRNA U62317.1 was significantly upregulated in OSCC samples compared with control samples, which is consistent with observed in the discovery dataset. Then each sample of GSE9844 was assigned a risk score according to 6lncRNAScore, and was classified as OSCC-like or normal-like samples. Finally, the 6lncRNAScore correctly classified all 26 OSCC samples and eight of 12 control samples, achieving an AUC of 0.968 with the sensitivity of 100% and the specificity of 66.7% (Figures 3B,C).

**FIGURE 4:**
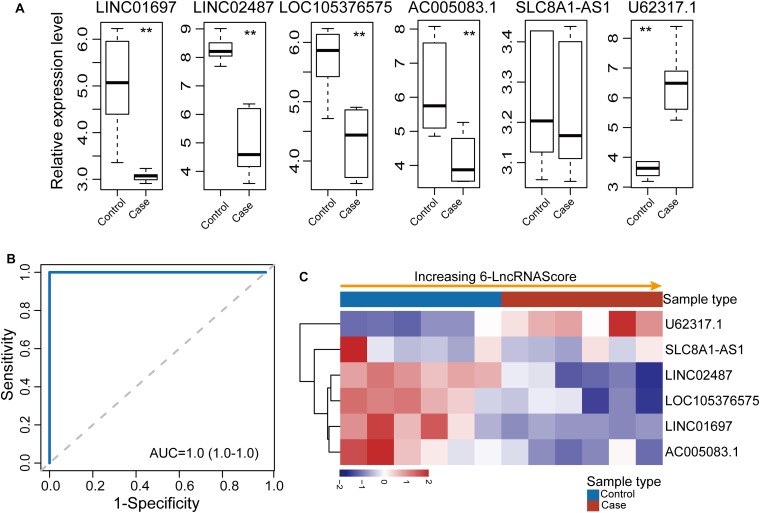
Further confirmation of the 6lncRNAScore in another GSE74530 dataset. **(A)** Expression patterns of six lncRNA biomarkers between OSCC samples and control samples. **(B)** Receiver operating characteristic (ROC) curves for 6lncRNAScore. **(C)** Expression heatmap of six lncRNA biomarkers of samples with increasing 6lncRNAScore. **p < 0.01.

Further validation of the diagnostic ability of the 6lncRNAScore was conducted using another completely independent OSCC dataset (GSE74530) of 12 samples. The expression pattern of six lncRNA biomarkers between OSCC samples and control samples in the GSE74530 dataset was examined. As shown in Figure 4A, five (*LINC01697*, *LINC02487*, *LOC105376575*, *AC005083.1*, and *U62317.1*) of six lncRNA biomarkers revealed consistent expression patterns as observed in the discovery dataset and GSE9844 dataset except for *SLC8A1-AS1*. When the 6lncRNAScore was tested in the GSE74530 dataset, the 6lncRNAScore correctly classified all six OSCC samples and three of six control samples, achieving an AUC of 1.0 with the sensitivity of 100% and the specificity of 50% (Figures 4B,C).

### Functional Implication of Six lncRNA Biomarkers

**FIGURE 5:**
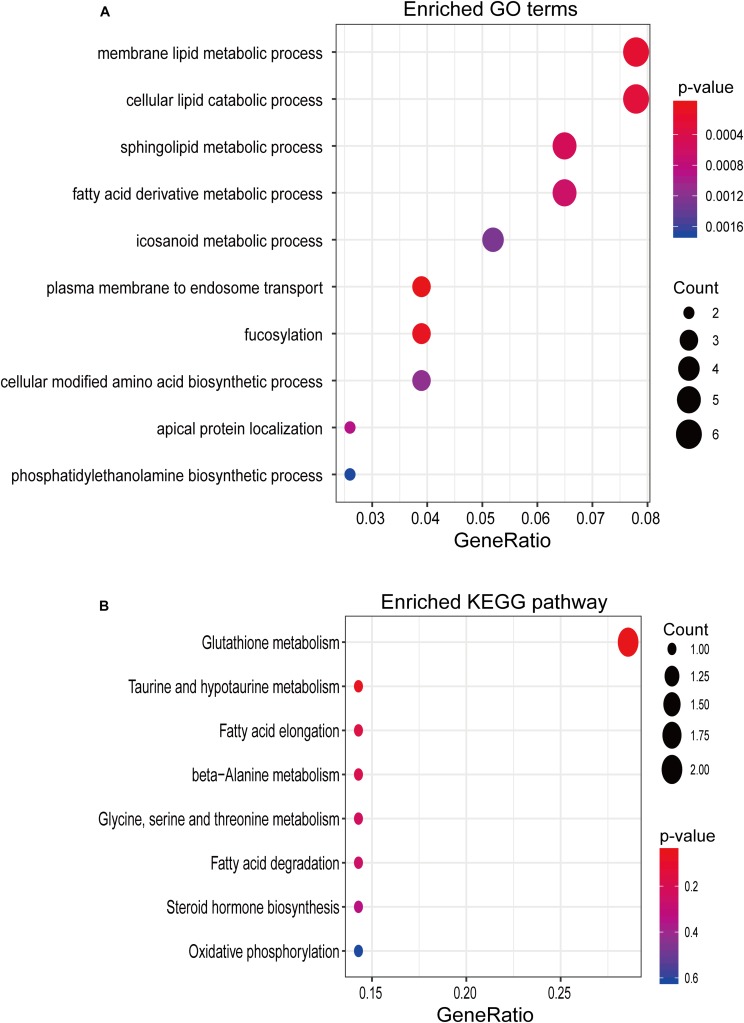
*In silico* functional analysis. **(A)** Enriched GO terms. **(B)** Enriched KEGG pathways.

To gain insight into the biological function of six newly identified lncRNA biomarkers, we computed the Pearson correlation coefficient (PCC) between expression levels of mRNAs and six lncRNA biomarkers and identified 95 mRNAs correlated with six lncRNA biomarkers (*r* > 0.8 and *p* < 0.05). Then we performed GO and KEGG enrichment analysis for 95 mRNAs correlated with six lncRNA biomarkers using the R package “clusterprofiler.” Results of functional enrichment analysis showed that mRNAs correlated with six lncRNA biomarkers might be involved in several lipid metabolic processes, such as membrane lipid metabolic process, cellular lipid catabolic process, sphingolipid metabolic process, fatty acid derivative metabolic process, and so on (Figure 5).

## Discussion

Although smoking and alcohol use were well-known chief risk factors for OSCC, complex genetic, transcriptional, and epigenetic alteration and their interaction between them have also been reported to indeed contribute to OSCC tumorigenesis (Choi and Myers, 2008; Perez-Sayans et al., 2009). Traditional diagnosis methods, such as the biopsy, endoscopy, and chest X-ray and CT, were inadequate for early diagnosis, and there is the lack of specific and sensitive biomarkers for earlier detection of OSCC. Alteration in molecular profiles have been observed in various human cancers compared to corresponding normal tissues, and have been used as novel markers for cancer diagnosis and prognosis (Mehta et al., 2010; Jurgensmeier et al., 2014; Chen et al., 2018, 2019; Sun et al., 2020). lncRNAs are recently emerging as a novel promising biomarker for cancer diagnosis and prognosis (Shi et al., 2016; Bolha et al., 2017). Increasing efforts have been made to focus on the discovery and characterization of lncRNA biomarkers in various human cancers. Recent studies also observed many lncRNAs dysregulation which served an oncogenic or tumor suppression part in OSCC (Lu et al., 2017; Kong et al., 2018; Zhang et al., 2019). Despite these previous investigations, the expression pattern and diagnostic role of lncRNA in OSCC remain unclear.

In this study, we first obtained lncRNA expression files of a large number of OSCC samples and corresponding control samples by repurposing publicly available microarray data, and compared the expression pattern of lncRNAs between OSCC and control samples as the first step toward identifying diagnostic lncRNA biomarkers. A total of 29 lncRNAs were determined as differentially expressed lncRNAs whose dysregulated expressions are closely associated with OSCC. To identify diagnostic lncRNA biomarkers from the list of differentially expressed lncRNAs, we performed a feature selection procedure to reduce the number of lncRNAs using the lasso binary logistic regression model which is the powerful and versatile regression method for high-dimensional data. Considering a balance between classification accuracy and the number of lncRNAs, six lncRNAs were identified as diagnostic biomarkers including five tumor suppressor lncRNAs (*LINC01697*, *LINC02487*, *LOC105376575*, *AC005083.1*, and *SLC8A1-AS1*) and one oncogenic lncRNAs (*U62317.1*). Although the huge number of lncRNAs have been identified using high-throughput experimental technologies, only a very small fraction of lncRNAs were well functionally characterized. After the literature search, we found that several lncRNA biomarkers identified in our study have been reported to be involved in human diseases. *LINC02487* has been reported as a tumor suppressor to inhibit migration and invasion of oral cancer cells through directly binding protein USP17 (Feng et al., 2019). lncRNA *AC005083.1* was also differentially expressed in lung adenocarcinoma (Peng et al., 2017). The latest study by Guo et al. found that LncRNA *SLC8A1-AS1* protects against myocardial damage through activation of the cGMP-PKG signaling pathway. Previous studies have suggested that lncRNA function can be inferred by using co-expression with the coding genes approach (Ma et al., 2012; Cheng et al., 2016). Therefore, to further explore the potential function of other lncRNA biomarkers, we performed functional enrichment analysis for lncRNA biomarkers-related mRNAs derived from co-expression analysis and found that mRNAs co-correlated with lncRNA biomarkers tended to be involved in lipid metabolic process. The modulation in lipid metabolism has been implicated in increased invasiveness of OSCC cells, and some lipid metabolism-related genes were used to predict poor outcome of OSCC (Beloribi-Djefaflia et al., 2016; Sant’Anna-Silva et al., 2018; Hu et al., 2019).

To accelerate clinical application, we constructed a diagnostic prediction model with these six lncRNA biomarkers using a logistic regression method. The 6lncRNAScore achieved an AUC value of 0.995 for distinguishing OSCC samples and healthy controls in the discovery dataset. To confirm the robustness of the 6lncRNAScore for early diagnosis, we also tested the performance of the 6lncRNAScore in the other two independent OSCC datasets. Validation results showed that the 6lncRNAScore also had well predictive performance in effectively discriminating OSCC patients from controls in the other two independent datasets comparable with the discovery dataset. These results demonstrated the reproducible and robust predictive power and general applicability of the 6lncRNAScore in early diagnosis of OSCC.

## Conclusion

In summary, we evaluated the utility of lncRNA expression in early diagnosis of OSCC and constructed a diagnostic prediction model composing of six lncRNA biomarkers which showed high and robust diagnostic performance in discriminating OSCC patients from controls. However, further experimental studies or independent validations are warranted to fully explore the molecular mechanism and clinical applications of lncRNAs in the diagnosis of OSCC.

## Data Availability Statement

Publicly available datasets were analyzed in this study. These data can be found here: Three publicly available OSCC datasets were obtained from the Gene Expression Omnibus (GEO) database (https://www.ncbi.nlm.nih.gov/geo/query/acc.cgi?acc=GSE30784, https://www.ncbi.nlm.nih.gov/geo/query/acc.cgi?acc=GSE9844, and https://www.ncbi.nlm.nih.gov/geo/query/acc.cgi?acc=GSE74530).

## Author Contributions

YL conceived and designed the experiments. YL, XC, and HL performed the experiments and analyzed the data. YL wrote the manuscript. All authors read and approved the final manuscript.

## Conflict of Interest

The authors declare that the research was conducted in the absence of any commercial or financial relationships that could be construed as a potential conflict of interest.

## Footnotes

1 https://www.ncbi.nlm.nih.gov/geo/

2 https://www.ncbi.nlm.nih.gov/geo/query/acc.cgi?acc=GSE30784

3 https://www.ncbi.nlm.nih.gov/geo/query/acc.cgi?acc=GSE9844

4 https://www.ncbi.nlm.nih.gov/geo/query/acc.cgi?acc=GSE74530
